# Antiviral Effects of ABMA against Herpes Simplex Virus Type 2 In Vitro and In Vivo

**DOI:** 10.3390/v10030119

**Published:** 2018-03-09

**Authors:** Wenwen Dai, Yu Wu, Jinpeng Bi, Shuai Wang, Fang Li, Wei Kong, Julien Barbier, Jean-Christophe Cintrat, Feng Gao, Daniel Gillet, Weiheng Su, Chunlai Jiang

**Affiliations:** 1 National Engineering Laboratory for AIDS Vaccine, School of Life Sciences, Jilin University, Changchun 130012, Jilin, China; daiwenwen92@163.com (W.D.); 15143080402@163.com (Ji.B.); a1251892522@163.com (S.W.); li296981502@126.com (F.L.); weikong@jlu.edu.cn (W.K.); feng0215@gmail.com (F.G.); 2 SIMOPRO, CEA, Université Paris-Saclay, F-91191 Gif Sur Yvette, France; wuyu81@gmail.com (Y.W.); Julien.BARBIER@cea.fr (Ju.B.); jean-christophe.cintrat@cea.fr (J.-C.C.); 3 Key Laboratory for Molecular Enzymology and Engineering of the Ministry of Education, School of Life Sciences, Jilin University, Changchun 130012, Jilin, China

**Keywords:** herpes simplex virus type 2, ABMA, antiviral effect, entry, late stage, vesicle transport

## Abstract

Herpes simplex virus type 2 (HSV-2) is the causative pathogen of genital herpes and is closely associated with the occurrence of cervical cancer and human immunodeficiency virus (HIV) infection. The absence of an effective vaccine and the emergence of drug resistance to commonly used nucleoside analogs emphasize the urgent need for alternative antivirals against HSV-2. Recently, ABMA [1-adamantyl (5-bromo-2-methoxybenzyl) amine] has been demonstrated to be an inhibitor of several pathogens exploiting host-vesicle transport, which also participates in the HSV-2 lifecycle. Here, we showed that ABMA inhibited HSV-2-induced cytopathic effects and plaque formation with 50% effective concentrations of 1.66 and 1.08 μM, respectively. We also preliminarily demonstrated in a time of compound addition assay that ABMA exerted a dual antiviral mechanism by impairing virus entry, as well as the late stages of the HSV-2 lifecycle. Furthermore, in vivo studies showed that ABMA protected BALB/c mice from intravaginal HSV-2 challenge with an improved survival rate of 50% at 5 mg/kg (8.33% for the untreated virus infected control). Consequently, our study has identified ABMA as an effective inhibitor of HSV-2, both in vitro and in vivo, for the first time and presents an alternative to nucleoside analogs for HSV-2 infection treatment.

## 1. Introduction

Genital herpes is one of the world’s most prevalent sexually transmitted diseases [1], and manifests as ulcerative and vesicular lesions on the genitals with lifelong latency [2]. Herpes simplex virus type 2 (HSV-2)—a single, large double-stranded DNA, enveloped virus belonging to the *Herpesviridae* family—is the major cause of genital herpes [3], and significantly increases the risk of developing cervical cancer and human immunodeficiency virus (HIV) infection [4,5,6]. HSV-2 infection is a global concern with estimates of 536 million people infected worldwide and an annual incidence of 23.6 million cases [7].

Despite the prevalence of infection in the global population, no vaccine has been developed and antiviral chemotherapy is standard practice in the management of HSV-2 infection [8,9]. However, long-term therapy with acyclovir and penciclovir as well as their prodrugs valaciclovir and famciclovir, respectively, has led to the emergence of drug resistance, especially in immune-compromised patients [10]. Additionally, various cases of toxicity have been encountered as a result of increasing use of traditional antivirals [11,12]. Although some non-nucleoside inhibitors have been developed, few are currently approved for HSV-2 infection treatment [13]. Foscarnet is approved as a second-line drug for HSV-2 infection treatment only when the patient has failed first-line treatment with acyclovir or there is a proven resistance mutation, and the use of foscarnet is limited by its toxicity and the fact that it is available only as an intravenous formulation [14]. Therefore, alternative antivirals against HSV-2 are needed.

The small molecule ABMA [1-adamantyl (5-bromo-2-methoxybenzyl) amine], was first identified from a cell-based high throughput screening, as an inhibitor of ricin, both in cell cultures and in mice, selectively acting on host-endosomal trafficking [15]. Subsequently, ABMA has been reported to be active against other infectious pathogens, including bacterial toxins (diphtheria toxin from Corynebacterium diphtheriae, lethal toxin from *Bacillus anthracis*, toxin B from *Clostridium difficile* and lethal toxin from *Clostridium sordellii*), viruses (*Ebola virus*, *Rabies virus* and *Dengue-4 virus*), bacteria (Simkaniaceae and Chlamydiaceae) and Leishmania parasite [15]. Each of these pathogens relies on host–endosomal trafficking for pathogenicity, indicating that the inhibitory effect of ABMA is related to host–vesicle transport [16,17,18].

HSV-2 initiates infection with attachment to host cells, followed by membrane fusion or endocytosis to enter the cells. Subsequently, de-enveloped tegument-capsids are transported to the nuclei, where genome transcription, DNA replication and new capsid assembly occur. Filled capsids then bud into vesicles derived from the trans-Golgi network to obtain an envelope and an outer membrane after release from the nuclei. Finally, viruses exit the host cells by fusion of enveloped virus-containing vesicles with the cell membranes. Several processes including endocytic virus entry, virus capsid envelopment and virus egress in the HSV-2 lifecycle depend on host–vesicle transport, providing a rationale for testing ABMA as an inhibitor of HSV-2.

In this study, we evaluated the antiviral activity of ABMA against HSV-2, in vitro and in vivo, and provided data to address possible mechanisms of action. Chloroquine, which was reported to inhibit herpes simplex virus infection by interacting with endocytic virus entry and the late stages of infection, as well as acyclovir, which is commonly used as the drug for HSV-2 infection treatment, were chosen as positive control drugs in this study [19,20,21,22,23,24]. ABMA was demonstrated to be an effective inhibitor of HSV-2 by a dual mechanism of action, acting on virus entry as well as the late stages of infection.

## 2. Materials and Methods

### 2.1. Cells, Virus, Compounds and Mice

African green monkey kidney cells (Vero cells) obtained from American type culture collection (ATCC) (cat # CCL-81) were cultured in Dulbecco’s modified Eagle’s medium (DMEM, Invitrogen, Carlsbad, CA, USA) supplemented with 10% fetal bovine serum (FBS, Invitrogen) (DMEM-10% FBS) at 37 °C with 5% CO_2_.

HSV-2 strain G obtained from ATCC (cat # VR-734) (KU310668) was propagated in Vero cells. Virus titration was performed by endpoint dilution and plaque assays.

**Figure 1:**
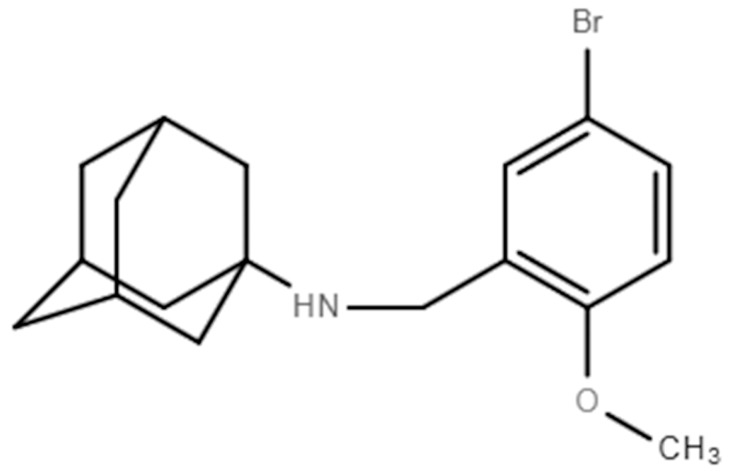
Chemical structure of ABMA.

ABMA [1-adamantyl (5-bromo-2-methoxybenzyl) amine] (Figure 1) was synthesized in-house. The positive control drugs, chloroquine and acyclovir, were purchased from Meilun Biotech Co., Ltd. (Dalian, China) [19,20,21,22,23,24]. The purities of the compounds were higher than 98%, as determined by High Performance Liquid Chromatography (HPLC). The compounds were dissolved in dimethyl sulfoxide (DMSO) as stock solutions.

Specific-pathogen-free female BALB/c mice (6–8 weeks old) were obtained from the Changchun Institute of Biological Products and maintained under the guidelines for animal experiments at Jilin University, China.

### 2.2. Cytotoxicity Assay

Cytotoxicity was measured by the Cell Titer-Glo^®^ Luminescent cell viability assay, as reported previously [25]. Serially diluted compounds were added to 90% confluent Vero cells in 96-well plates. After incubation for 72 h, cell viability was assayed with the Cell Titer-Glo^®^ reagent (Promega, Madison, WI, USA) and quantified by the PerkinElmer VICTORTM X2 (Waltham, MA, USA). Cytotoxicity was measured by the percentage of the luminescence intensity of compound-treated cells relative to that of the untreated cell control. The 50% cytotoxicity concentration (CC_50_) was calculated by regression analysis of the dose–response curves [26].

### 2.3. Antiviral Activity Assay of ABMA against HSV-2 In Vitro

The antiviral activity of ABMA against HSV-2 was measured by cytopathic effect (CPE) inhibition and plaque reduction assays. In the CPE inhibition assay, serially diluted ABMA or chloroquine (positive control drug), was added to 90% confluent Vero cells in 96-well plates 5 h before infection with HSV-2 (MOI = 0.04, which was determined to cause appropriate CPE (100%) and cell viability reduction (60%) on Vero cells after infection for 72 h), while acyclovir (positive control drug) was added at the same time as infection. After incubation for 72 h post-infection in the presence of the compounds, cell viability was measured as described in Section 2.2.

In the plaque reduction assay, serially diluted ABMA or chloroquine (positive control drug), was added to Vero cell monolayers in 12-well plates 5 h before infection with HSV-2 (50–100 PFU, which was determined to ensure that appropriate numbers of plaques form in the plates and are counted accurately), while acyclovir (positive control drug) was added at the same time as infection. After infection for 1 h, DMEM-2% FBS-1% low-melting agarose containing the above compounds at corresponding concentrations was overlaid in place of infected medium. Plaque numbers were counted after the cells were fixed with 4% paraformaldehyde and stained with 0.5% crystal violet when plaques formed.

CPE inhibition and plaque reduction rates were measured by the following equations: CPE inhibition (%)=(T−V)(C−V)×100%, where C, V and T are the luminescence intensities of the untreated cell control, the untreated virus infected control and compound-treated cells, respectively. Plaque reduction (%)=[1−(plaque number)T(plaque number)V]×100%, where (plaque number)_T_ and (plaque number)_V_ are plaque numbers of compound-treated cells and the untreated virus-infected control, respectively. The 50% effective concentration (EC_50_), which refers to the concentration of a drug that induces a response halfway between the baseline and the maximum after a specified exposure time, was calculated by regression analysis of the dose–response curves [26].

### 2.4. Western Blotting

Cell samples were lysed in RIPA buffer (Beyotime Biotech Co., Ltd., Shanghai, China) and the lysates were cleaned by centrifugation at 12,000 rpm. The proteins were separated by SDS-PAGE and transferred onto nitrocellulose membranes. After being blocked with 3% non-fat milk for 1 h, the membranes were incubated with an anti-HSV-2 VP5 (major capsid protein of HSV-2 [27]) mouse monoclonal antibody (EastCoast Bio, North Berwick, ME, USA) or an anti-β-tubulin mouse monoclonal antibody (Covance, Emeryville, CA, USA) for 2 h. Blots were subsequently incubated with an alkaline phosphatase (AP)-conjugated anti-mouse IgG antibody (SouthernBiotech, Birmingham, AL, USA) for 1 h and developed by the interaction between AP and AP substrates, followed by termination with the exposure to light.

### 2.5. Quantitative Polymerase Chain Reaction (qPCR) Assay

The Ezup Column Virus DNA Purification Kit (Sangon Biotech Co., Ltd., Shanghai, China) was used to extract HSV-2 DNA. TransStart^®^ Top Green qPCR SuperMix (TransGen Biotech Co., Ltd., Beijing, China) and gG specific primers (forward primer: 5′-CCCACACCCCAACACATC-3′, reverse primer: 5′-CCAAGGCGACCAGACAAAC-3′) were used for amplification and subsequent quantification with the Bio-Rad CFX96 system (Hercules, CA, USA).

### 2.6. Time of ABMA Addition Assay

In the antiviral activity assay against HSV-2 based on the measurement of viral protein and DNA content in the cell cultures, 3.13 μM ABMA or 15 μM chloroquine was added to 90% confluent Vero cells in 24-well plates 5 h before infection with HSV-2 (MOI = 1, which was determined to ensure synchronized infection in a single replicative lifecycle as reported [28]), while 1 μM acyclovir was added at the same time as infection. After infection for 1 h, DMEM-2% FBS containing the above compounds at their corresponding concentrations was overlaid in place of the medium. Proteins and HSV-2 DNA were extracted and quantified as described in Section 2.4 and Section 2.5 at 18 h post-infection, when a single lifecycle had been completed without the occurrence of obvious CPE [29].

In the effective stage assay, ABMA (3.13 μM) and HSV-2 (MOI = 1) were added to 90% confluent Vero cells in 24-well plates following different treatment schemes, as reported with some modifications [29]. To study a prophylactic effect (pre: −5–0 h), the cells were pretreated with ABMA for 5 h, then infected with HSV-2 after removal of ABMA by washing. To study an inhibitory effect on virus binding or entry (simultaneous: 0–1 h), the cells were treated with ABMA and infected with HSV-2 at the same time, then overlaid with DMEM-2% FBS after removal of the medium by washing at 1 h post-infection. To study the effect on virus replication (early post: 1–6 h), the infected cells were treated with ABMA from 1 h to 6 h post-infection, then overlaid with DMEM-2% FBS after removal of ABMA by washing. To study the effect on late stage infection (late post: 6–18 h), the infected cells were cultured with DMEM-2% FBS for 5 h, then treated with ABMA from 6 h to 18 h post-infection. Besides, HSV-2 was pre-incubated with ABMA at 4 °C for 5 h before infection (direct) to study the direct interactions in a cell free system. HSV-2 infection was performed during 0–1 h for all procedures, except for direct procedure, in which the cells were infected with HSV-2 that had been pretreated with ABMA. HSV-2 DNA in the cell cultures was extracted and quantified as described in Section 2.5 at 18 h post-infection.

### 2.7. Binding and Entry Assays

Binding and entry assays were performed as reported previously, with some modifications [30]. The 90% confluent Vero cells in 24-well plates were pretreated with 3.13 μM ABMA, 15 μM chloroquine or 1 μM acyclovir for 5 h before addition of HSV-2. In the binding assay, the cells were exposed to HSV-2 (MOI = 1) at 4 °C for 2 h. Unbound viruses were removed by washing twice with sterile PBS buffer at 4 °C, and HSV-2 DNA from the original virus inoculum and the unbound virus supernatant were extracted separately and quantified as described in Section 2.5 to calculate the amount of bound HSV-2.

In the entry assay, the cells were further incubated at 37 °C for 1 h after the binding process. After two freeze–thaw cycles of the infected cells, HSV-2 DNA from the internalized virus was extracted and quantified to calculate the amount of HSV-2 that was able to enter the cells.

### 2.8. Late Stage Infection Assay

To study the effects of ABMA on the late stages of the HSV-2 lifecycle, 90% confluent Vero cells in 24-well plates were infected with HSV-2 (MOI = 1) for 1 h, then treated with 3.13 μM ABMA, 15 μM chloroquine or 1 μM acyclovir during 6–18 h post-infection. At 18 h post-infection, the supernatants and the infected cells were collected and subjected to direct extracellular virus titration and to intracellular virus titration after two freeze–thaw cycles. Virus titers were determined by the Reed and Muench dilution method and expressed as 50% tissue culture infectious doses per milliliter (TCID_50_/mL).

### 2.9. Antiviral Efficacy Assay of ABMA against HSV-2 In Vivo

Female BALB/c mice (6–8 weeks old, *n* = 10–12 per group) were injected subcutaneously with 2 mg of Depo-Provera (XianJu Pharmaceutical Co., Ltd., Taizhou, China) per mouse to induce a diestrus phase in the genital tract. Seven days later, the mice were inoculated intravaginally with 50,000 PFU of HSV-2 in 10 μL of PBS after anesthesia. At 1 h post-inoculation, and subsequently once daily for seven consecutive days, 1.25 mg/kg or 5 mg/kg of ABMA (the doses were determined to ensure sufficient dissolution of ABMA in the injections), or 150 mg/kg of acyclovir (positive control) [31] was administered intraperitoneally. The compounds were all dissolved in PBS supplemented with 10% DMSO and PBS supplemented with 10% DMSO was administered as an untreated virus infected control. The mice were monitored daily for survival rate and clinical score. Signs of disease were evaluated as: 0, healthy; 1, genital erythema; 2, moderate genital inflammation; 3, genital lesion; 4, hind-limb paralysis; 5, death [32]. Vaginal swab samples were collected at day 5 and day 10 and transferred to 200 μL of Hank’s buffer. HSV-2 titers from the swab samples were determined by plaque assay in Vero cells as reported [33]. Protocols for animal experiments were approved by the Committee on Animal Experimental Ethics of School of Life Sciences at Jilin University [permission code: 2017-nsfc019, 15 January 2017].

### 2.10. Statistical Analysis

In vitro experiments were conducted in technical triplicate and repeated three times independently. A one-way ANOVA test was used for statistical analysis to compare the differences between test groups and untreated virus infected control groups. A log-rank test (Mantel–Cox) was used for comparisons of the survival curves. Statistical significance is represented by asterisks and was marked correspondingly in the figures (* *p* < 0.05, ** *p* < 0.01, *** *p* < 0.001).

## 3. Results

### 3.1. Reductions of HSV-2-Induced Cytopathic Effects and Plaque Formation Were Detected in ABMA-Treated Cells

**Figure 2:**
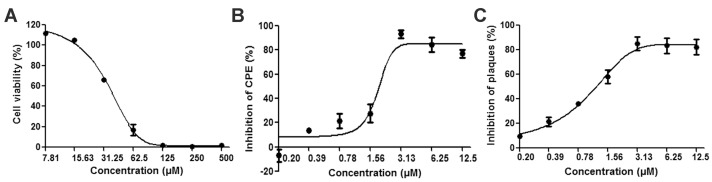
Cytotoxicity and antiviral activity of ABMA against HSV-2 in vitro. (**A**) Serially diluted ABMA was added to Vero cells, then cell viability was measured and compared to that of the untreated cell control after incubation for 72 h. (**B**) Serially diluted ABMA was added to Vero cells 5 h before infection with HSV-2 (MOI = 0.04), then cell viability was measured to calculate the CPE inhibition percentage after infection for 72 h in the presence of ABMA. (**C**) Serially diluted ABMA was added to Vero cells 5 h before infection with HSV-2 (50–100 PFU) for 1 h, then DMEM-2% FBS-1% low-melting agarose containing ABMA at corresponding concentrations was overlaid in place of infected medium, followed by counting of plaque numbers to calculate the plaque inhibition percentage when plaques formed.

**Figure 3:**
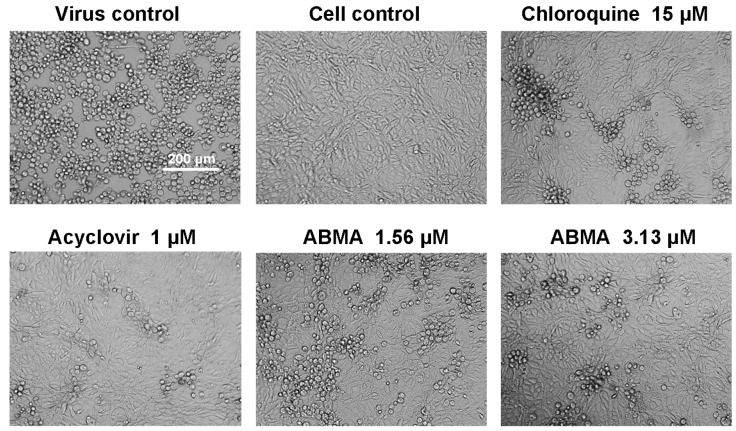
Effects of compounds on HSV-2-induced CPE. Morphological changes of Vero cells were recorded by phase-contrast microscopy at 72 h post-infection following the CPE inhibition assay. Virus control and cell control represented untreated virus infected cells and uninfected cells, respectively. Scale bar = 200 μm.

ABMA was tested for cytotoxicity before assessing its antiviral activity. As shown in Figure 2A and Table 1, Vero cells responded to ABMA in a dose-dependent manner with a CC_50_ value of 34.75 μM. No cytotoxicity was observed at the concentrations effective against HSV-2 infection. It is current practice in drug discovery processes to pretreat cells with the compound before infection, in order to better monitor a positive effect. ABMA was tested for anti-HSV-2 activity with treatment administered from 5 h before infection to the end of the assays, as reported previously [15]. As shown in Figure 2B and Table 1, ABMA inhibited HSV-2-induced CPE in a dose-dependent manner with an EC_50_ value of 1.66 μM and a maximum inhibition rate of 93.36% at 3.13 μM. The selective index (SI) measuring the safety of a compound to be developed as an antiviral agent was calculated to be 20.93 by CC_50_ relative to EC_50_ [34], which was higher than that of the positive control drug, chloroquine. A plaque reduction assay was performed subsequently to confirm the anti-HSV-2 activity. As shown in Figure 2C and Table 1, ABMA inhibited HSV-2-induced plaque formation in a dose-dependent manner with an EC_50_ value of 1.08 μM, which was in accordance with the results obtained in the CPE inhibition assay. Morphological changes of the cells also confirmed the protective effects of ABMA against HSV-2 infection. As shown in Figure 3, untreated virus-infected cells appeared all rounded up and detached from the plates, uninfected cells looked all spread out, while virus infected cells treated with the drugs were mostly spread out and partially rounded up. Thus, ABMA is a safe and effective antiviral agent against HSV-2 in vitro, which protects cells from HSV-2 infection below its toxic concentration.

### 3.2. Reductions of HSV-2 Protein and DNA Content Were Detected in ABMA-Treated Cells

**Figure 4:**
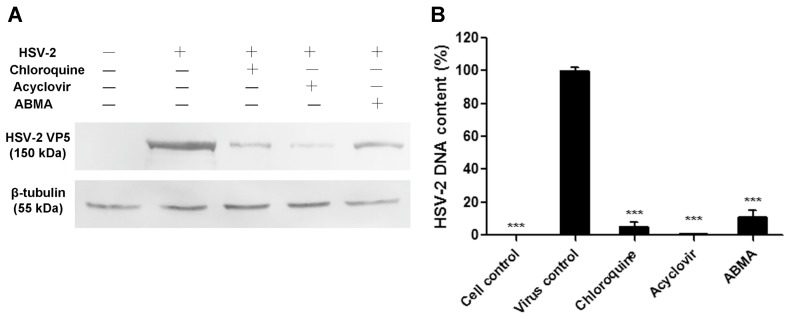
Effects of compounds on HSV-2 protein and DNA content in cell cultures. 3.13 μM ABMA or 15 μM chloroquine was added to Vero cells 5 h before infection with HSV-2 (MOI = 1), while 1 μM acyclovir was added at the same time as infection. Then DMEM-2% FBS containing the compounds at their corresponding concentrations was overlaid in place of the medium after infection for 1 h. (**A**) Proteins were extracted from the cell cultures at 18 h post-infection, then separated by SDS-PAGE and analyzed by Western blot using an anti-HSV-2 VP5 antibody or an anti-β-tubulin antibody. “+” and “−” represented “with” and “without” the additions, respectively. (**B**) HSV-2 DNA was extracted from the cell cultures at 18 h post-infection and quantified by qPCR. Statistical significance was compared between test groups and the untreated virus infected control group and was represented by asterisks marked in the figures, where *** *p* < 0.001.

The effects of ABMA on HSV-2 proliferation were measured by quantifying HSV-2 protein and DNA content in the cell cultures. ABMA treatment was administered from 5 h prior to infection, through to the end of the assays. HSV-2 protein and DNA content were assayed by Western blot and qPCR assays, respectively, after a single replicative cycle, before the occurrence of obvious CPE at 18 h post-infection [29]. As shown in Figure 4A, a significant reduction in the content of HSV-2 VP5 (main capsid protein of HSV-2 [27]) was observed in ABMA-treated cells, while that of β-tubulin (constitutive protein essential for cell function) was not affected. As shown in Figure 4B, a significant reduction in HSV-2 DNA content was also detected in ABMA-treated cells. The positive control drugs, chloroquine and acyclovir reduced HSV-2 protein synthesis and DNA replication as expected [19,20,21,22,23,24]. Based on those data, ABMA appears to be an effective antiviral agent against HSV-2 in vitro, which can cause reduced HSV-2 protein and DNA content in the cell cultures.

### 3.3. ABMA Blocks HSV-2 Entry into Cells

**Figure 5:**
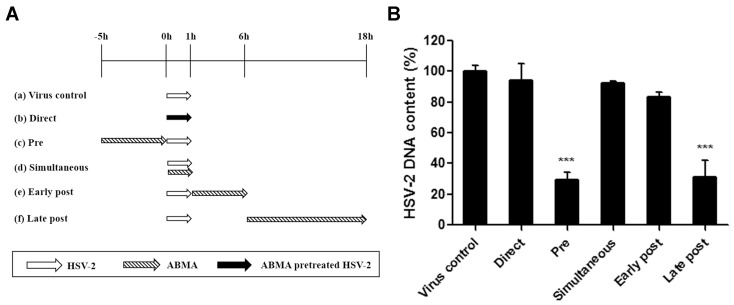
Effects of ABMA on different stages of the HSV-2 lifecycle. (**A**) ABMA (3.13 μM) treatment and HSV-2 (MOI = 1) infection schemes in the time of ABMA addition assay. (**B**) HSV-2 DNA was extracted from the cell cultures at 18 h post-infection following the treatment schemes in (**A**) and quantified by qPCR. Statistical significance was compared between test groups and the untreated virus infected control group and was represented by asterisks marked in the figures, where *** *p* < 0.001.

The effects of ABMA on different stages of the HSV-2 lifecycle were measured by a mode of action assay following different ABMA treatment schemes (Figure 5A). ABMA was added at time points corresponding to different events in the HSV-2 lifecycle. HSV-2 DNA content in the cell cultures for all assay conditions were measured at 18 h post-infection. Significant reductions in HSV-2 DNA content were detected when the cells were pre-treated with ABMA prior to infection (pre), or when ABMA was introduced from 6–18 h post-infection (late post) (Figure 5B). The results strongly suggested that ABMA affected the early events in the HSV-2 lifecycle by acting on the cells directly. ABMA also had an effect on the late stages of the HSV-2 lifecycle as a result of treatment during 6–18 h post-infection (Figure 5B). As there was no loss in cell viability at the concentration of ABMA used in the experiments (3.13 μM) (Figure 2A), the inhibitory effects of ABMA were not due to cytotoxicity. Therefore, ABMA affects both early and late stages of the HSV-2 lifecycle. The latter mechanism was discussed further in Section 3.4.

**Figure 6:**
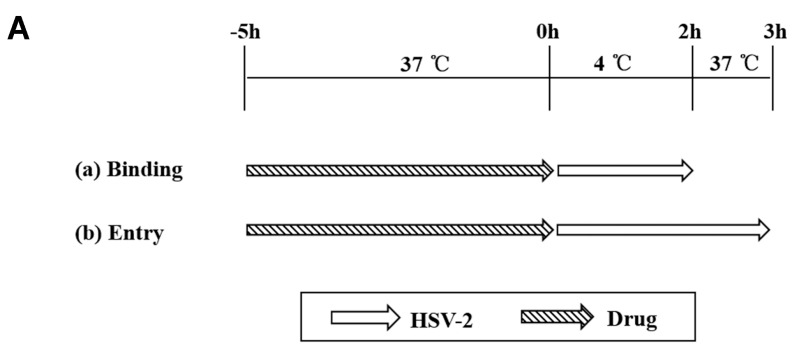

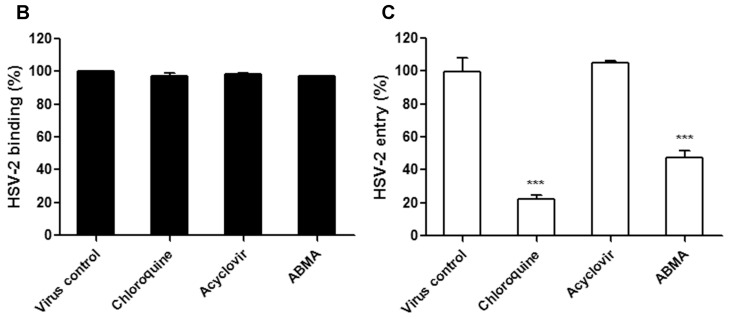
Effects of compounds on HSV-2 binding and entry. (**A**) Drug treatment and HSV-2 (MOI = 1) incubation schemes in binding and entry assays. (**B**) HSV-2 DNA was extracted from the original virus inoculum and unbound virus supernatant at 2 h post-incubation of the cells with HSV-2 at 4 °C and quantified to calculate the amount of HSV-2 bound to the cells in the binding assay. (**C**) HSV-2 DNA was extracted from the internalized virus after two freeze-thaw cycles of the infected cells at 1 h post-infection with HSV-2 at 37 °C following the binding process and quantified to calculate the amount of HSV-2 that was internalized in cells in the entry assay. Statistical significance was compared between test groups and the untreated virus infected control group and was represented by asterisks marked in the figures, where *** *p* < 0.001.

As antiviral agents targeting the essential early stages of the HSV-2 lifecycle (binding and entry) may be more effective than those targeting the late stages, events in the early stages of the HSV-2 lifecycle that could be targeted by ABMA were further investigated [35]. HSV-2 binding and entry assays were performed at 4 °C and 37 °C, respectively, after pretreatment of the cells with the compounds. This was followed by quantification of bound and entered viruses by measuring HSV-2 DNA content in the original virus inoculum, the unbound virus supernatant from the binding assay (unbound virus) and the internalized virus after freeze–thaw cycles of the infected cells from the entry assay (entered virus), respectively (Figure 6A). As shown in Figure 6B,C, HSV-2 entry was significantly reduced by ABMA, similarly to chloroquine, which is known to affect HSV-2 entry [20,21,24]. As expected, acyclovir that blocks virus replication had no effect on virus binding or entry [19]. Therefore, ABMA blocks HSV-2 entry into cells.

### 3.4. ABMA Inhibits the Late Stages of the HSV-2 Lifecycle

**Figure 7:**
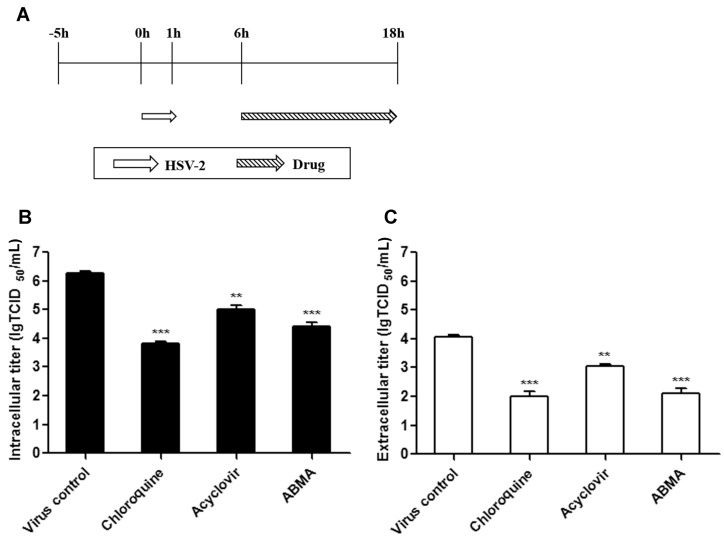
Effects of compounds on the late stages of the HSV-2 lifecycle. (**A**) Drug treatment and HSV-2 (MOI = 1) incubation schemes in the late stage infection assay. (**B**) Intracellular viruses were collected from the infected cells after two freeze-thaw cycles at 18 h post-infection following the treatment schemes in (**A**) and subjected to virus titration. (**C**) Extracellular viruses were collected from the cell supernatants at 18 h post-infection following the treatment schemes in (**A**) and subjected to virus titration. Statistical significance was compared between test groups and the untreated virus infected control groups and was represented by asterisks marked in the figures, where ** *p* < 0.01, *** *p* < 0.001.

The effects of ABMA on the late stages of the HSV-2 lifecycle were further studied using the late stage infection assay at 18 h post-infection. HSV-2-infected cells were treated with the compounds during 6–18 h post-infection, which corresponds to the late stages of the HSV-2 lifecycle. Following this, intracellular and extracellular virus titers were measured (Figure 7A). As shown in Figure 7B, both intracellular and extracellular HSV-2 titers were significantly reduced by ABMA. As chloroquine was reported to interact with the late stages of the HSV lifecycle, virus titers were significantly reduced, as expected [20,22,23]. As the target of acyclovir was demonstrated to be HSV-2 DNA replication, which mainly takes place during 3–6 h post-infection, virus titers were reduced to a lesser extent [19]. These results confirmed the inhibitory effects of ABMA on the late stages of the HSV-2 lifecycle, as described in Section 3.3. Additionally, these results also suggested that the HSV-2 packaging and egress process was most likely to be blocked by ABMA, as capsid formation and progeny infectious particle packaging gradually take place from 5 h post-infection onwards [36].

### 3.5. ABMA Protects BALB/c Mice from Intravaginal HSV-2 Challenge

**Figure 8:**
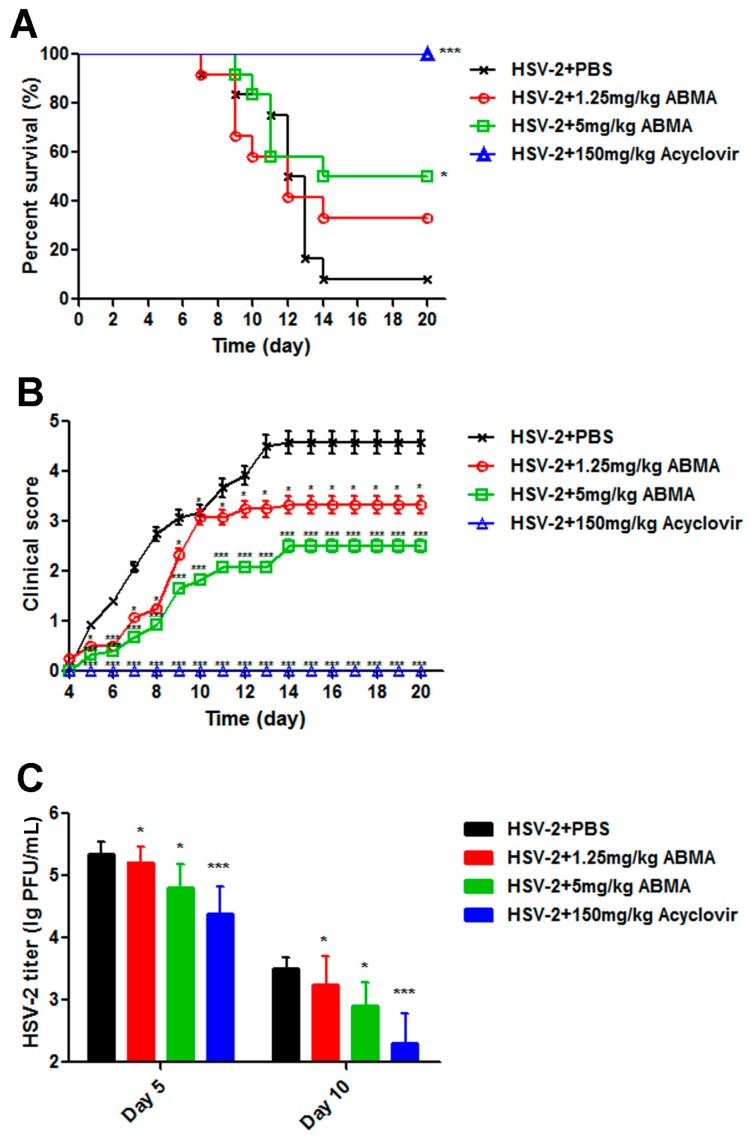
Antiviral efficacy of ABMA against intravaginal HSV-2 challenge in BALB/c mice. Female BALB/c mice (6–8 weeks old, *n* = 10–12 per group) were injected subcutaneously with 2 mg of Depo-Provera per mouse seven days before intravaginal inoculation with 50,000 PFU of HSV-2 in 10 μL of PBS. At 1 h post-inoculation and once daily for seven consecutive days, 1.25 mg/kg or 5 mg/kg of ABMA, 150 mg/kg of acyclovir, or PBS supplemented with 10% DMSO was administered intraperitoneally. (**A**) Survival rate and (**B**) clinical score of the mice were monitored daily for 20 days. (**C**) Vaginal swab samples were collected at day 5 and day 10, respectively, and transferred to 200 μL of Hank’s buffer, followed by virus titer determination by plaque assay in Vero cells. Statistical significance was compared between test groups and the untreated virus infected control and was represented by asterisks marked in the figures, where * *p* < 0.05, *** *p* < 0.001.

Having identified the anti-HSV-2 potency of ABMA in vitro, we next evaluated the protective efficacy of ABMA against intravaginal challenge of HSV-2 in BALB/c mice using a reported mouse model [37]. As shown in Figure 8A, ABMA significantly improved the survival rates of drug-treated infected mice compared to the untreated virus infected control, whose survival rate was 8.33%. ABMA given daily intraperitoneally at 5 mg/kg provided the best survival rate of 50%. As shown in Figure 8B, ABMA at both doses tested reduced the clinical score in the same trend as the survival rate. Mice treated with 5 mg/kg of ABMA showed the lowest clinical score, below 3, while the value of the untreated virus infected control reached 4.58. Several mice with clinical score below 2 (moderate genital inflammation) recovered from the infection along with time. However, mice with clinical score higher than 2 did not recover. As clinical scores in Figure 8B were presented as the mean value of 10–12 mice, there was no reduction in clinical scores along with time as a result. Despite that, ABMA significantly reduced the severity of the disease and slowed the progress time course of mice compared to the untreated virus infected mice. As acyclovir is normally used as the standard treatment of HSV-2 infection, it was used as the positive control drug in the in vivo experiment [31]. The protective rate of acyclovir at 150 mg/kg was shown to be 100%, as expected [31]. Body weight changes of mice were also recorded over 20 days, but there was no significant difference among these groups (data no shown). HSV-2 titers from the vaginal swabs at day 5, when viral load reached its peak, and at day 10, for a later time point, were also detected to confirm the protective efficacy of ABMA against HSV-2 infection in vivo [38]. As shown in Figure 8C, HSV-2 was detected in all groups indicating a successful HSV-2 challenge. ABMA at 5 mg/kg significantly reduced HSV-2 titers by 0.55 log and 0.60 log at day 5 and day 10, respectively. Acyclovir at 150 mg/kg significantly reduced HSV-2 titers by 0.97 log and 1.20 log at day 5 and day 10, respectively. Based on these data, ABMA effectively protects BALB/c mice from intravaginal HSV-2 challenge.

Overall, ABMA is an effective antiviral agent against HSV-2 in vitro and in vivo, with two putative modes of action, affecting both virus entry and the late stages of the HSV-2 lifecycle.

## 4. Discussion

The prevalence, severity of complications and the close association with cervical cancer and HIV infection make HSV-2 infection a global health concern [39]. The lack of an available vaccine and the emergence of drug resistance highlight the importance of developing alternative antivirals against HSV-2 with distinct modes of action [10]. ABMA has been demonstrated previously to be active against several intracellular pathogens exploiting host–vesicle transport [15]. Here we demonstrated that ABMA is an effective inhibitor of HSV-2, in vitro and in vivo.

The anti-HSV-2 potential of ABMA was first identified in vitro with EC_50_ values below 2 μM and an SI value of 20.93, which is suitable for an antiviral agent [40] (Figure 2 and Table 1). Treatment with ABMA also resulted in significant reductions of HSV-2 protein and DNA content in the cell cultures (Figure 4). Subsequently, ABMA was found to target both early and late stages of the HSV-2 lifecycle in the time of compound addition assay, which was similar to the effects of SPL-2999 (a dendrimer with the active surface group of naphthyl 3,6-disulfonic acid sodium salts to interact with biological surfaces) on HSV-2 [41] (Figure 5).

ABMA was found to affect the early stages of HSV-2 infection by acting on cells directly, as reported in previous studies on the effects of polysaccharide extracts from algal species and SPL-2999 on HSV-2 [41,42]. The specific event targeted by ABMA in the early stages of HSV-2 infection was found to be virus entry into cells, while binding was unaffected by ABMA treatment, which was similar to the effect of Dynasore (a small-molecule inhibitor of dynamin, which is a GTPase that controls multiple endocytic pathways and also plays a role in actin assembly and reorganization) on HSV-2 entry [30] (Figure 6). Although herpes simplex virus (HSV) may enter cells by direct fusion between the virus envelope with the cell membranes, substantial evidences for HSV entry through an endocytic-dependent mechanism have come to light [43]. HSV begins endocytic entry by using the host membrane machinery to envelop viruses. The viruses trapped in endocytic vesicles can then be released to the host cytoplasm by fusion of the virus envelope with the vesicle membranes [44]. The inhibitory effect of ABMA on HSV-2 entry might be related to its effect on host–vesicle transport [15], which is involved in the endocytic entry pathway of HSV-2. Antivirals targeting early stage infection have attracted significant attention because reduced entry of viruses into cells translates to decreased replication and spread to other cells [35]. Most early-stage prevention drugs that show promise in treating HSV-2 infection are binding inhibitors, targeting the host cell receptor or the viral glycoprotein required for binding [43,44]. Agents preventing HSV-2 entry into cells, including SPL-2999, PM-19 (a keggin-type heteropolyoxotungstate K_7_[PTi_2_W_10_O_40_]·6H_2_O), Dynasore and ABMA reported in our study may provide alternative early stage inhibitors [30,41,45].

In addition to virus entry, ABMA was also found to inhibit the late stages of HSV-2 infection, which was confirmed by significantly reduced intracellular and extracellular virus titers when ABMA was applied during 6–18 h post-infection (Figure 7). Additionally, because HSV-2 DNA replicates rapidly between 3–6 h and the formation of both the capsids and the progeny infectious particles of HSV-2 gradually takes place from approximately 5 h post-infection onwards [36], the results also suggested that ABMA was most likely to hinder the HSV-2 packaging and egress process, as reported in a study on the effects of Nelfinavir on HSV-1 [46]. The HSV-2 packaging and egress process begins with capsid assembly in the nuclei, then infectious particles are packaged by budding the capsids into specialized vesicles derived from the trans-Golgi network to gain an envelope and an outer vesicular membrane. Finally, the virus-containing vesicles move to and fuse with the cell membranes to release the viruses into the extracellular medium [47,48,49]. The fact that ABMA was likely to block the HSV-2 packaging and egress process suggested two possible explanations: the target of ABMA present on host–endosomal transport is also present on the HSV-2 packaging and egress process. Alternatively, host–endosomal transport, which can be affected by ABMA, participates in the HSV-2 packaging and egress process.

Significant protective efficacy of ABMA against intravaginal HSV-2 challenge in female BALB/c mice was demonstrated by an improved survival rate, reduced clinical score and reduced vaginal virus load compared to the untreated virus infected control (Figure 8). ABMA administered at the highest tested dose of 5 mg/kg showed the best survival rate of 50%, while that of the untreated virus infected control was 8.33%. Several recently developed inhibitors of HSV-2 have undergone, or are currently undergoing clinical trials, but most of them have yet to gain licensure due to adverse effects on the host [13]. Based on our study, ABMA might provide an alternative. As ABMA is an initial hit from a high throughput screening, further medicinal chemistry and pharmaceutical optimizations are still required and may lead to candidates with higher anti-HSV-2 activities.

Multi-drug therapy, combining drugs with different modes of action to limit the manifestation of drug resistance and to increase the selectivity by reduced dose, is common practice in the treatment of viral infections, including HIV and HCV (hepatitis C virus), but not HSV, for which currently available drugs have the same target, the viral DNA polymerase [50,51]. ABMA was demonstrated to be an effective inhibitor of HSV-2 with the targets of virus entry as well as the late stages of viral lifecycle, which were different from commonly used acyclovir. It might well be possible to develop combinations of ABMA with acyclovir, or other drugs targeting stages different from ABMA in the HSV-2 lifecycle, such as virus replication. These novel combinations might be more effective and might provide an alternative to HSV-2 infection treatment.

In conclusion, AMBA has been identified as an effective inhibitor of HSV-2 in vitro and in vivo, by inhibiting virus entry, as well as the late stages of the HSV-2 lifecycle. Our study expands the list of pathogens against which ABMA is active and exemplifies the potential of ABMA to be developed as a broad-spectrum inhibitor. As the target of ABMA is a host component rather than the pathogens themselves, drug resistance may be less likely to arise [52].

## Figures and Tables

**Table 1:** Cytotoxicity and antiviral activity of compound against HSV-2 in Vero cells.

	Cytotoxicity	Antiviral Activity		
Compounds	CC_50_ (μM)	EC_50_^a^ (μM)	EC_50_^b^ (μM)	SI ^c^
ABMA	34.75 ± 0.28	1.66 ± 0.14	1.08 ± 0.25	20.93
Chloroquine	15.67 ± 0.47	1.86 ± 0.20	1.38 ± 0.03	8.42
Acyclovir	>1000	0.82 ± 0.06	0.81 ± 0.07	1219.51

^a^ Concentration at which the compound CPE inhibition rate reaches halfway between the baseline and the maximum; ^b^ concentration at which the compound plaque reduction rate reaches halfway between the baseline and the maximum; ^c^ selective index (SI) value represents the ratio of CC_50_/EC_50_^a^ for each compound; Results are presented as mean values ± standard deviations (SD) obtained from three independent experiments.
